# Diagnostic Prediction for Social Anxiety Disorder via Multivariate Pattern Analysis of the Regional Homogeneity

**DOI:** 10.1155/2015/763965

**Published:** 2015-06-09

**Authors:** Wenjing Zhang, Xun Yang, Su Lui, Yajing Meng, Li Yao, Yuan Xiao, Wei Deng, Wei Zhang, Qiyong Gong

**Affiliations:** ^1^Huaxi MR Research Center (HMRRC), Department of Radiology, West China Hospital, Sichuan University, Chengdu 610041, China; ^2^School of Sociality and Psychology, Southwest University for Nationalities, Chengdu 610041, China; ^3^Radiology Department of the Second Affiliated Hospital, Wenzhou Medical University, Wenzhou, Zhejiang 325027, China; ^4^Department of Psychiatry, West China Hospital, Sichuan University, Chengdu 610041, China

## Abstract

Although decades of efforts have been spent studying the pathogenesis of social anxiety disorder (SAD), there are still no objective biological markers that could be reliably used to identify individuals with SAD. Studies using multivariate pattern analysis have shown the potential value in clinically diagnosing psychiatric disorders with neuroimaging data. We therefore examined the diagnostic potential of regional homogeneity (ReHo) underlying neural correlates of SAD using support vector machine (SVM), which has never been studied. Forty SAD patients and pairwise matched healthy controls were recruited and scanned by resting-state fMRI. The ReHo was calculated as synchronization of fMRI signals of nearest neighboring 27 voxels. A linear SVM was then adopted and allowed the classification of the two groups with diagnostic accuracy of ReHo that was 76.25% (sensitivity = 70%, and specificity = 82.5%, *P* ≤ 0.001). Regions showing different discriminating values between diagnostic groups were mainly located in default mode network, dorsal attention network, self-referential network, and sensory networks, while the left medial prefrontal cortex was identified with the highest weight. These results implicate that ReHo has good diagnostic potential in SAD, and thus may provide an initial step towards the possible use of whole brain local connectivity to inform the clinical evaluation.

## 1. Introduction

Previously termed social phobia, social anxiety disorder (SAD) was characterized by persistent fear of social or performance situations in which there is judgment or scrutiny by others [1]. As the most common anxiety disorder, SAD shows a high lifetime prevalence of 12% and a 12-month prevalence of 7.1% [2]. Early onset, delay, or avoidance in seeking treatment leads to significant social and occupational disability for individuals with SAD. Currently, according to the diagnostic criteria in DSM-IV (Diagnostic and Statistical Manual of Mental Disorders, Fourth Edition), the diagnosis of SAD is based on observed behaviors and examinations of psychiatric signs and symptoms. However, the use of such a symptom-based approach would sometimes cause uncorrected diagnosis due to high rates of comorbidity with depressive conditions and substance abuse [3]. Therefore, it is necessary to establish other objective and reliable approaches or biological markers, which could be used to assist the diagnosis of SAD and improve the accuracy.

As a more objective approach, neuroimaging holds great promise for detecting abnormalities crucial to the pathophysiologic models of SAD. Resting-state functional magnetic resonance imaging (fMRI) studies have revealed that, relative to the healthy controls, SAD is associated with abnormal activation within amygdala and default model network (DMN) mainly involved [4–6]. The amygdala is thought to be important in the acquisition and expression of conditioned fear and also performs a protective role, allowing the organism to detect and avoid danger [4], whilst role of DMN may be relevant to social perception and self-referential processing which are underlying psychological symptoms and pathophysiological mechanisms in SAD [6, 7]. These findings are valuable in helping us understand the functional changes which underlie clinical symptoms associated with SAD; however, the extrapolation of any potential biomarkers and the clinical translation of the results have been hindered by the group level inference of the data. For the imaging findings to be clinically useful, one must be able to make inferences at the individual rather than the group level.

Relative to traditional univariate methods, multivariate pattern analysis (MVPA) allows predictions individually and it takes the patterns of information that might be presented across multiple variables into account, therefore providing results that have higher translational applicability in clinical practice [8]. For fMRI data, MVPA involves whole brain pattern classification aimed at decoding information in the pattern of activation across all voxels that may distinguish between two classes at the individual level. The most commonly used MVPA for pattern recognition in neuroimaging literature is support vector machine (SVM) [9]: an algorithm uses a well-defined dataset to create decision function or “hyperplane” which can best distinguish between categories (in current study, patients and controls), and then the produced decision function or “hyperplane” will be used to predict which predefined group a new observation belongs to. These two phases are systematically known as training and testing [9]. The overall accuracy of the SVM depends on its sensitivity (i.e., the proportion of patients identified as having the disease) and specificity (i.e., the proportion of controls identified as not having the disease). In recent years, SVM algorithm has been successfully applied to classify various neuropsychiatric disorders and achieved good diagnostic accuracy [10].

To date, there have been only two studies using MVPA in SAD but concentrating on task-based fMRI and regional grey matter volume [11], or functional connectivity [7]. As reflected by resting-state fMRI, functional connectivity can reveal the synchronization of remote brain regions, while, by contrast, regional homogeneity (ReHo) has been developed to measure the local synchronization of spontaneous fMRI signals by calculating similarity of dynamic fluctuations of voxels within a given cluster, revealing important information about local connectivity, and reflects the temporal synchrony of the regional fMRI BOLD signals [12, 13]. Abnormal ReHo is assumed to be associated with aberrant changes in the temporal aspects of the spontaneous neural activity in the regional brain [14] and may be a sign of disrupted local functionality [15]. More importantly, ReHo can indicate some unexpected hemodynamic responses that model-driven methods may fail to discover in resting-state fMRI [13]. Although being successfully applied to various neuropsychiatric disorders [14, 16–20], the ReHo approach has been little investigated in SAD.

Thus, we particularly used SVM to examine ReHo maps in differentiating SAD patients from healthy controls, which has never been investigated. The purposes were to find out whether SVM would allow accurate discrimination between diagnostic groups and, if so, which brain regions or intrinsic brain networks would principally contribute to the discrimination.

## 2. Materials and Methods

### 2.1. Participants

Forty Structured Interview for the DSM-IV (SCID) Patient Edition confirmed SAD patients and an equal number of healthy controls were recruited at the Mental Health Centre of West China Hospital (Table 1). The Ethics Committee of West China Hospital, Sichuan University, has offered approval to our study and all participants gave written informed consent to their participation. Diagnosis of SAD was determined by consensus of two experienced psychiatrists. Psychological ratings and clinical symptoms associated with SAD were evaluated with the Liebowitz Social Anxiety Scale (LSAS). Of the 40 patients, 12 had the antianxiety medication but they underwent at least two-week washing-out prior to the MR examination.

Healthy controls were recruited from the local area via poster advertisements and were screened using the SCID-Non-Patient Version to ascertain the lifetime absence of psychiatric and neurological illness. It was confirmed that they had no history of psychiatric illness among their first-degree relatives. All subjects' demographic characteristics and clinical variables were obtained by 2 experienced clinical psychiatrists before MR examinations. Patients with SAD and control subjects were pairwise matched in age, gender, and handedness (Table 1). The following exclusion criteria applied to both groups: (1) the existence of a neurological disorder or other psychiatric disorders, (2) substance abuse, (3) pregnancy, or (4) major physical illness such as cardiovascular disease or hepatitis, as assessed by clinical evaluations and medical records. T1-weighted and T2-weighted images of brain were inspected by an experienced neuroradiologist, and no scanning artifacts and gross abnormalities were observed in any participants.

### 2.2. MRI Acquisition

The MRI examinations were performed on a whole-body 3.0 T MR scanner (Siemens Trio, Erlangen, Germany) with a 12-channel head coil. Earplugs were employed to protect the hearing while foam pads upon them were used to restrict head motion during the scanning. The resting-state fMRI sensitized to changes in BOLD signal levels was obtained with a gradient-echo planar imaging sequence (TR/TE = 2000/30 ms; flip angle = 90°). A field of view (FOV) of 240 × 240 mm^2^ was used with an acquisition matrix = 64 × 64, producing 30 continuous axial slices with thickness = 5.0 mm with no gap and voxel size = 3.75 × 3.75 × 5 mm^3^ in-plane resolution in each brain volume. Each functional run contained 205 volumes of which the first 5 were discarded to ensure steady-state longitudinal magnetization and subjects' adaptation to the environment. All participants were simply instructed to keep still with their eyes closed and remain awake but not to think of anything in particular. After the scanning, the volumes of all subjects were corrected for the temporal difference and head motion by setting the translational or rotational parameters at the threshold of ±1.5 mm or ±1.5°.

### 2.3. Imaging Preprocessing

The fMRI data was preprocessed using Data Processing Assistant for Resting-State fMRI (DPARSF, http://www.restfmri.net, version 2.1), implemented within the MATLAB toolbox, to calculate the ReHo maps. This software involves an integrated image process mainly including slice timing, realignment, and normalization to the Montreal Neurological Institute echo planar imaging template (each voxel was resampled to 3 × 3 × 3 mm^3^), removing linear trend and the ReHo calculation. Given the fact that ReHo shows the similarity or synchronization of fMRI signals of nearest neighboring voxels and Kendall's coefficient of concordance (KCC) is used for the measurement based on the regional homogeneity hypothesis [13], we defined 27 nearest neighboring voxels as a cluster and a KCC value was given to the voxel at the center of this cluster. The individual ReHo map was generated in a voxel-wise fashion, and all ReHo maps were smoothed with a Gaussian filter of 4 mm full-width half maximum (FWHM) kernel to manage the anatomical variability that was not compensated for by spatial normalization.

### 2.4. Comparison of Demographic Characteristics and Variables

The Statistical Package for the Social Sciences (SPSS, version 18.0) will be used for the comparison of demographic variables. Differences in age and LSAS scores between groups were analyzed using the two-sample *t*-tests, whereas gender ratio was compared with Chi-square test, with significance levels setting at *P* < 0.05.

### 2.5. Multivariate Pattern Analysis and Support Vector Machine

SVM as implemented in the PROBID software package (http://www.brainmap.co.uk/probid.htm, version 1.04) was employed and a linear kernel SVM was adopted to classify the diagnostic groups based on their ReHo maps. The detailed description of the application of SVM in MRI data has been given [8, 21]. In the context of supervised multivariate classification method as SVM [22], individual brain scans were treated as points located at high-dimensional space defined by the ReHo map in the preprocessed images. In this high-dimensional space, a linear decision boundary was defined by a “hyperplane” that separated the individual brain scans according to a class label (i.e., patients versus controls). The optimal hyperplane was computed based on the whole multivariate pattern of ReHo map across each image and could most accurately capture the relationship between each example and its respective label. The algorithm is initially trained on a subset of the data 〈*x*, *c*〉 to find a hyperplane that best separates the input space according to the class labels *c* (patients versus controls), where *x* represents the input data (i.e., ReHo map). The linear kernel SVM adopted could reduce the risk of overfitting the data and allow direct extraction of the weight vector as an image (i.e., the SVM discrimination map). Furthermore, the linear kernel matrix implicated in PROBID could be precomputed and supplied to the classifier, an approach which affords a substantial increase in computational efficiency and permits whole brain classification without requiring explicit dimensionality reduction [23]. A parameter *C*, which controls the tradeoff between having zero training errors and allowing misclassifications in the linear model, was fixed at *C* = 1 for all cases (default value). A grey matter mask of 3 × 3 × 3 mm was used to constrain the search of significant group differences in voxels/features within grey matter in the comparison of ReHo maps.

Consistent with previous studies using SVM on SAD [7, 11], a “leave-one-out” cross validation was used, which means a single subject of each group would be excluded from the training and was later used to test the capability of the classifier learned from the remaining subjects, to reliably distinguish between categories (in our study, SAD or controls). Each subject pair would undergo this procedure to make the accuracy of the SVM fully estimated [8]. Statistical significance of the overall classification accuracy was determined by permutation testing [24, 25], a nonparametric test that involved repeating the classification procedure 1000 times with a different random permutation of the training group labels and counting the number of permutations achieving higher sensitivity and specificity than the true labels. Finally, to show the multivariate discriminating pattern of ReHo maps, a threshold would be set at 30% of the maximum weight vector value of the discrimination and voxels with greater value would be exhibited.

The test margin (the shortest distance from the optimal hyperplane), which could show the capability of the SVM with ReHo in classification of each subject, was calculated for all participants. Based on the label and test margin of each subject, the receiver operating characteristic (ROC) curve of the classification with ReHo maps was obtained with SPSS. To further explore whether the classification is driven by anxious symptoms and the extent if so, correlation analysis has been performed between the test margin and the level of symptom severity as determined by LSAS scores for all participants.

## 3. Result

### 3.1. Demographic and Clinical Characteristics

Demographic and clinical characteristics for all of participants are presented in Table 1. No significant differences were found in gender ratio and age between patients and healthy controls (*P* > 0.05). Compared to healthy control, SAD patients had significantly higher scores on the anxiety symptoms measured with LSAS total score and subscales (*P* < 0.05). All participants were right-handed. Twenty-eight patients were drug-naïve while the remaining 12 had taken different medication (5 paroxetine, 3 paroxetine with intermittent risperidone, alprazolam, and buspirone, resp., 3 sertraline, and 1 amitriptyline and doxepin) for 1 week to 5 years. The medicated patients had been drug-free for at least 2 weeks.

### 3.2. Multivariate Pattern Recognition

**Figure 1:**
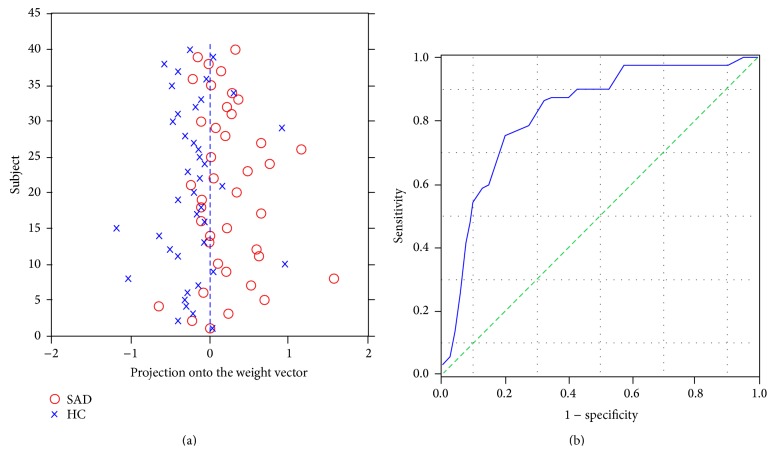
Classification plot (a) obtained from PROBID and receiver operating characteristic (ROC) curve and (b) obtained from SPSS for the discrimination between SAD patients and healthy controls using ReHo maps, yielding an accuracy of 76.25% (sensitivity = 70.0% and specificity = 82.5%, *P* ≤ 0.001).

**Figure 2:**
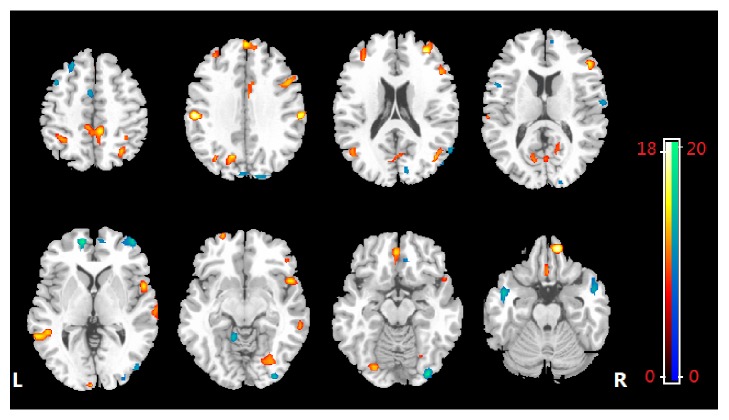
The discrimination maps for ReHo. Regions displayed were identified by setting the threshold to ≥30% of the weight vector scores. Warm color (positive value) indicated higher discriminated values in SAD than in healthy controls; cool color (negative weights) indicates higher values in healthy controls than in SAD.

The classification of the two groups with overall diagnostic accuracy of ReHo maps was 76.25% (sensitivity = 70% and specificity = 82.5%, *P* ≤ 0.001) achieved by SVM (Figure 1). The set of regions showed different value between the diagnostic groups mainly located in frontal, temporal, and occipital regions (Figure 2, Table 2). In the discrimination map, a positive value means a relative higher weight in SAD (red scale) and helps in the identification of individuals with SAD, with regions mainly located at right orbitofrontal gyrus (OFG), right middle frontal gyrus, right pars triangularis, right superior temporal gyrus (STG), left middle temporal gyrus (MTG), right postcentral gyrus (PCG), left inferior parietal lobe (IPL), and right precuneus, while a negative value means a relative higher weight in healthy controls and contributes to the identification of healthy subjects, locating in left medial prefrontal cortex (mPFC), bilateral middle frontal gyrus (MFG), right inferior occipital gyrus (IOG), and right cuneus (Figure 2).

### 3.3. Relationship between Test Margin and Severity of Symptom

Across all of the patients, the test margin was found not correlated to total LSAS scores, scores for fear factor, or scores for avoidance factor (*P* > 0.05).

## 4. Discussion

To the best of our knowledge, the current study is the first to examine the capability of SVM with ReHo in distinguishing patients with SAD from healthy subjects and involves the largest sample of SAD patients in employing MVPA approach. By identifying the intergroup differences in whole brain ReHo pattern with an overall classification accuracy of 76.25%, the present study suggests local connectivity and synchronization extracted from fMRI BOLD signal could be a potential biomarker to identify SAD patients and demonstrates that multivariate analysis allows discrimination between individuals with SAD and healthy controls at relatively high level of accuracy. This pattern of results provides preliminary support to the development of SVM as a promising diagnostic tool in SAD to improve the diagnostic accuracy and minimize errors in detecting malingering where possible [26].

The discriminating pattern in the present study was attributable to widespread ReHo alterations, mainly involving DMN, dorsal attention network (DAN), self-referential network (SRN), and sensory networks. By contrast, the only one study using ReHo in SAD before found significantly decreased ReHo mainly in the DMN and central executive network (CEN) while it found increased ReHo in occipital regions and the right putamen in a relative small sample of SAD patients via mass-univariate analysis [27]. Relatively, our findings consistently revealed abnormalities within DMN but identified more regions, and other distributed regions across brain have also been showed with different local connectivity between SAD and control subjects. One explicable fact is that MVPA implicated in SVM takes the interregional correlation into account [8]. This multivariate nature of SVM rendered a high discriminative power for a given cluster deriving not only from differences in ReHo in that region between groups, but also from any intergroup differences in its functional correlations with other regions. Thus, the findings of the altered ReHo across brain should not be deemed as individual regions but as a spatially distributed pattern. Taken collectively, ReHo investigation gives insight into coherent local connectivity of a functional cluster and is necessary for further interpreting functional changes in SAD patients, whilst the combination with SVM identifies more distributed and subtle ReHo changes helpful in characterization at individual level therefore yielding results with great potential in clinical translation.

The consistent finding of altered local connectivity in relation to DMN emphasizes its critical role underlying the pathogenesis of SAD. The DMN is deemed as a higher-level cognitive network and consists of brain regions that typically activate during resting-state but deactivate during performance of goal-directed tasks [28], within which a set of regions connectively contributes to the social cognitive aspects. The precuneus, along with posterior cingulate cortex (PCC), is featured as the pivotal hub of DMN and related to perception of social cognition and self-related mental representations [28, 29]. Patients with SAD showed a lower deactivation in regions comprising precuneus during task conditions [30] and abnormal functional connectivity in precuneus has also been suggested to be associated with the pathophysiological mechanism underlying SAD [31]. The mPFC is another hub in DMN and is identified with the highest weight. Activity in mPFC may reflect an interaction between cognitive processing and emotional state [32], especially for the anxiety-related emotion processing, in which mPFC has been considered of ongoing importance [33]. These data, along with our findings, supported the notion that DMN accounts for prominence in cognitive behavioral models of SAD.

DAN [34, 35] and SRN [36] are another two important networks with many regions found with alteration with ReHo in SAD patients. DAN is considered to mediate goal-directed (top-down) processing for stimuli selection and responses and involved in many higher-order cognitive tasks [34]. Particularly emotion regulation, the regulating of anxious feelings, has been emphasized in the cognitive models underlying SAD [37], and failure in emotion regulation has been considered another key feature of SAD [38]. In this term of view, we speculate that there is an important role of brain regions within DAN in emotion regulation, and abnormalities in these regions usually result in a high level of self-awareness, typically in SAD patients. While the SRN has exhibited peculiar physiological characteristics with increased neural activity during resting [28], OFC is a primary region observed with local coherence alteration. As involved in the engagement of interpersonal relationships, moral behavior, and social aggression [39–41], OFC with inappropriate function might strengthen the response to stressors or stimuli of fear conditions, resulting in severe impairments in social behavior [5]. Reduced orbitofrontal activation was observed in patients with SAD during public speaking [42] and anxiety-provoking tasks [43], implying a hypoactive OFC was associated with a failure of fear and anxiety inhibition.

There are still main regions implicated in visual network (VN), auditory network (AN), and somatomotor network (SMN). However, sensory networks could be regarded as the lower-order system of cognition. Within VN, abnormalities in IOG may be associated with the hypervigilance and hyperprosexia characteristic of social interaction in SAD [44]. Additionally, together with SMN, VN has been suggested to show significantly greater BOLD responses in SAD for social threat in previous emotional fMRI study [45]. As for AN, regions within which have been found to related to dysfunction of cognitive reappraisal in SAD patients [46]. Consistent with these findings, our results imply a role for sensory networks in the perceptual and some other psychological impairments in SAD to variable extents.

Although the discriminating pattern above successfully allowed the identification of patients with SAD, the accuracy was not that high to achieve the goal of MVPA of automated MR image analysis in finding better sensitivity and specificity of antemortem diagnosis than what is currently possible [47]. While the performance, generalizability, and significance of the SVM findings would benefit from a large sample size and better feature selection methods [48], future studies incorporating large sample are needed to improve characterization of underlying features, as to establishment of a model which could most accurately predict new subjects for better classification. Furthermore, expanding feature selection to include other imaging properties, behavioral data, and genomic information may offer better discriminative information for predicting SAD.

In the exploratory analysis, no significant association was found between the test margin and clinical symptoms. In other words, the distance away from the hyperplane may not be driven or affected by the severity of symptoms as assessed by LSAS scores for a given subject, suggesting the discriminating pattern of ReHo obtained is relatively stable. This may be because the discrimination pattern produced derived mostly from the intergroup ReHo differences free from clinical ratings, which is of great significance since the identification of SAD will not be confounded by the psychological situations, reducing the rate of false negative findings resulting from individuals with mild symptoms.

It is noteworthy that there are some limitations implicated in the present study. First, although most of patients were drug-naïve, a small proportion of the SAD sample had taken medication before. However, we have prepared two weeks for the washing-out before scanning to reduce the confounding effect resulting from medication. Besides, given the effect of antianxiety medication in attenuating abnormally activated neural activity in social anxiety [49], we thus speculated that the medication effect would probably not exaggerate but instead tend to underestimate the capability of SVM in identifying patients. That might also be the reason why the present study did not find the abnormal alterations in amygdala due to attenuated amygdala responsiveness [50]. Second, as we have added whole voxels in the grey matter in the pattern analysis, the intrinsic structural differences may act as confounding factors in the pattern recognition analysis. However, ReHo and structural properties are different features in the SVM analysis; since we used the functional features implicated in ReHo, the confounding influence of structural differences was assumed marginal, if there were any. While we did not have sufficient structural images to conduct the same analysis to rule out confounding factors, future studies with different imaging modalities will be needed as a synthesized biomarker to strengthen the classification and achieve more reliable clinical diagnosis of this complex disorder. Finally, as a common psychiatric disorder, social anxiety has a potential correlation but differs from a personality trait known as shyness. While in current study, we only compare the cohort of SAD patients with healthy subjects, leaving an issue unresolved whether the application of SVM to ReHo would also discriminate SAD patients from mentally healthy people with shyness. The future studies may help to address this question by including a third group of subjects who have a level of shyness but without SAD.

## 5. Conclusion

This study used a MVPA method which is based on whole brain ReHo pattern, to distinguish individuals with SAD from healthy subjects. By presenting widespread differential map of coherence abnormalities which could be used to identify patients with SAD at the individual level, this study provides evidence that the ReHo of brain has the diagnostic potential and can possibly act as a supplementary approach to identify SAD, especially regions with high weight. Future studies with the integration of ReHo with other different imaging modality measurements may give a better insight into the imaging biomarkers of the condition.

## Figures and Tables

**Table 1:** Demographic and clinical characteristics of SAD patients and healthy comparison subjects.

Demographic and clinical characteristics	SAD (*n* = 40)		HC (*n* = 40)		*P*
	Mean	SD	Mean	SD	
Age (years)	25.95	6.48	24.80	3.35	0.323
Duration of illness (years)	7.76	4.61			
Age of onset (years)	18.15	6.70			
LSAS					
Total	65.42	21.23	33.80	22.01	0.000
Fear factor	32.45	10.39	15.70	11.90	0.000
Avoidance factor	32.47	11.91	17.85	11.34	0.000

	*N*	%	*N*	%	*P*

Gender					
Female	14	35	14	35	1.000
Male	26	65	26	65	

SAD, social anxiety disorder; HC, healthy controls; SD, standard deviation; LSAS, Liebowitz Social Anxiety Scale; *n*/*N*, number.

Significance levels were set at *P* < 0.05.

**Table 2:** Regions of ReHo map discriminating between individuals with SAD and control subjects. These regions were exhibited by setting the threshold to ≥30% of the maximum weight vector value.

SAD > HC					SAD < HC				
Regions	*x*	*y*	*z*	*w*_*i*_	Regions	*x*	*y*	*z*	*w*_*i*_
Frontal lobe					Frontal lobe				
Left superior frontal gyrus	−23	64	−8	11.29	Left medial superior frontal gyrus	−11	56	5	−19.03
Right medial superior frontal gyrus	9	51	35	9.55	Right medial superior frontal gyrus	10	58	5	−11.89
Left medial superior frontal gyrus	1	53	35	12.16	Left superior frontal gyrus	−3	3	53	−13.96
Right middle frontal gyrus	33	47	23	16.54	Right middle frontal gyrus	45	57	2	−16.49
	50	14	32	13.19	Left middle frontal gyrus	−39	14	48	−13.32
Left middle frontal gyrus	−31	42	35	10.85		−25	33	51	−13.64
Right inferior frontal gyrus	48	37	−9	8.65	Left inferior frontal gyrus	−48	10	13	−8.1
Right pars triangularis	51	33	13	12.6					
Left medial orbitofrontal gyrus	−2	46	−14	13.04					
Right orbitofrontal gyrus	12	49	−22	17.88					
Right precuneus	7	−40	54	17.73					
	17	−58	13	9.07					
Temporal lobe					Temporal lobe				
Right superior temporal gyrus	56	9	−1	14.13	Right middle temporal gyrus	52	8	−24	−13.16
	68	−21	3	11.14	Left temporal pole	−45	3	−19	−14.59
Right middle temporal gyrus	63	−37	−10	8.38	Right postcentral gyrus	66	−9	14	−12.53
	48	−64	0	8.63					
Left superior temporal gyrus	−60	−26	11	10.26					
Left middle temporal gyrus	−44	−63	17	8.79					
	−58	−47	2	13.92					
Occipital lobe					Occipital lobe				
Left middle occipital gyrus	−33	−71	33	10.99	Right inferior occipital gyrus	35	−88	−15	−17.29
Right fusiform gyrus	24	−74	−10	9.96		49	−80	−1	−14.91
Left cuneus	−2	−101	5	12.31	Right cuneus	15	−82	23	−14.57
Left cuneus	−11	−71	13	10.05	Left cuneus	−2	−87	35	−11.1
Left lingual gyrus	−24	−81	−16	13.19					
Parietal lobe					Cerebellum				
Right superior parietal gyrus	32	−62	50	10.75	Left cerebellum	−9	−47	−9	−14.75
Left inferior parietal gyrus	−53	−22	35	17.59					
	−33	−47	50	9.3					
Left precuneus	−13	−67	32	12.02					
Right postcentral gyrus	63	−23	32	16.56					

SAD, social anxiety disorder; HC, healthy controls.

*w*_*i*_: weight of each cluster centroid, the value which indicates the relative contribution to the classification.
